# Prevalence and Determinants of Poor Sleep Quality Among Patients with Established Coronary Heart Disease in Kazakhstan: A Multi-Center Cross-Sectional Study

**DOI:** 10.3390/medsci14040439

**Published:** 2026-07-27

**Authors:** Kairat Davletov, Dimash Davletov, Bekbolat Zholdin, Mukhtar Kulimbet, Dinmukhammed Osser, Gulnara Kurmanalina, Vadim Medovchshikov, Nurlan Yeshniyazov, Farida Ibragimova, Alisher Makhmutov, Leylan Abdullaeva, Marat Pashimov, Batyrbek Assembekov

**Affiliations:** 1 Population Health Research Center, Al-Farabi Kazakh National University, Almaty 050012, Kazakhstan; davletov.k@kaznmu.kz; 2 Atchabarov Scientific-Research Institute of Fundamental and Applied Medicine, Asfendiyarov Kazakh National Medical University, Almaty 050040, Kazakhstan; velievaleylan@gmail.com (L.A.); assembekov.b@kaznmu.kz (B.A.); 3 Department of Internal Diseases No. 2, West Kazakhstan Marat Ospanov Medical University, Aktobe 030000, Kazakhstan; bekbolat.zholdin@gmail.com (B.Z.); gulnara.kurmanalina@mail.ru (G.K.); medovchshikov@zkmu.kz (V.M.); yeshniyazovn@gmail.com (N.Y.); 4 Research Institute of Cardiology and Internal Diseases, Almaty 050000, Kazakhstan; mkbkul@gmail.com (M.K.); iii.kz@mail.ru (F.I.); pashimov.marat@bk.ru (M.P.); 5 Danbury Hospital/Northwell, Danbury, CT 06810, USA; dinmukhammedosser@gmail.com; 6 Ascension Saint Joseph Hospital, Chicago, IL 60657, USA; alshain9@gmail.com

**Keywords:** coronary heart disease, sleep quality, secondary prevention, PSQI, Kazakhstan, ischemic heart disease

## Abstract

Background: Poor sleep quality is an increasingly recognized, modifiable cardiovascular risk factor, yet data among patients with established coronary heart disease (CHD) in Central Asia are scarce. We estimated the prevalence of poor sleep quality and identified its correlates in a Kazakhstani CHD population. Methods: In this multi-center cross-sectional study, 398 patients 6–24 months after an index coronary event were assessed for sleep quality. Sleep quality was measured with the Pittsburgh Sleep Quality Index (PSQI; >5 = poor). Anxiety and depression, physical activity, cognition, and clinical and socio-demographic variables were recorded. Associations were examined using logistic regression with subgroup and interaction analyses. Results: Poor sleep quality was reported by 147 patients (36.9%). In the adjusted model, anxiety was the only independent factor associated with poor sleep (OR 1.88, 95% CI: 1.10 to 3.23). The subgroup analysis suggested anxiety (OR 3.94) and lower education (OR 2.58) as correlates in women, whereas retirement (OR 2.27) and low physical activity (OR 2.31) were linked to poor sleep in men. A significant sex–anxiety interaction demonstrated a weaker anxiety effect in men. Conclusions: More than one-third of CHD patients reported poor sleep quality, driven primarily by anxiety, with distinct sex- and age-specific determinants. Sleep and psychological screening should be integrated into secondary prevention, with personalized approaches.

## 1. Introduction

Cardiovascular diseases (CVD) remain the leading cause of mortality and disability worldwide, with ischemic heart disease being the largest single contributor to global cardiovascular death [1,2]. More than three-quarters of the global CVD burden falls on low- and middle-income countries, including the Central and Eastern European and Central Asian regions [3,4]. In Kazakhstan, CVD causes roughly half of all deaths. The country’s age-standardized cardiovascular mortality is approximately double the global average, and ischemic heart disease is the leading cause of death [3,5,6,7].

Survivors of acute coronary events and patients with chronic coronary heart disease (CHD) are at substantial risk for recurrent cardiovascular events, and effective secondary prevention requires concurrent control of medical, behavioral, and psychosocial risk factors [8,9]. Despite well-established guidelines, large multinational surveys such as EUROASPIRE IV and V have repeatedly demonstrated that the majority of CHD patients fail to achieve lifestyle, risk factor, or therapeutic targets, with persistent smoking, physical inactivity, obesity, and inadequate blood pressure or lipid control [10,11,12,13]. Less than half of European coronary patients access cardiac prevention and rehabilitation programs, and lifestyle modification has been shown to be more successful in those who do [10,12].

Sleep has emerged as a key, modifiable component of cardiovascular health. In 2022, the American Heart Association added healthy sleep duration as the eighth metric of its Life’s Essential 8 framework [14]. Both short and long sleep duration, and poor subjective sleep quality, have been associated with an increased incidence of CHD, stroke, and cardiovascular and all-cause mortality [15,16,17,18]. Insomnia in particular has been linked to incident myocardial infarction, recurrent major adverse cardiovascular events, and long-term mortality after acute myocardial infarction [19,20,21,22,23]. Plausible mechanisms include sympathetic hyperactivity, hypothalamic–pituitary–adrenal axis dysregulation, low-grade systemic inflammation, endothelial dysfunction, and disturbed circadian regulation of blood pressure and glucose [24,25,26,27].

Sleep, anxiety, and depression share strong bidirectional relationships, and all three are highly prevalent in patients with established CHD [28,29,30,31]. Reported prevalence rates of poor sleep quality among cardiovascular patients range widely from approximately 33% to over 70%, depending on population, setting, and instrument used [32,33,34].

Anxiety, depression, and post-traumatic stress disorder (PTSD) are common after acute coronary events. Pooled prevalence estimates after acute myocardial infarction reach approximately 28.7% for depression and 5.5–58.2% for anxiety, with PTSD reported in roughly 12% of acute coronary syndrome patients [35,36,37]. Both depression and anxiety in CHD patients have been independently linked to increased cardiovascular and all-cause mortality [38,39,40,41]. Sleep disturbance frequently coexists with these psychological conditions and may serve as a shared mechanism linking them to cardiovascular outcomes [31].

Despite growing recognition of sleep as a modifiable cardiovascular risk factor, data on the prevalence and determinants of poor sleep quality among patients with established CHD remain limited in Central Asia and, to our knowledge, have not been systematically reported in Kazakhstan. Given the cultural, demographic, and health system differences between Central Asia and the predominantly European or East Asian populations in which this question has been studied, locally relevant evidence is needed to guide secondary prevention strategies [3]. Therefore, this study aimed to (a) estimate the prevalence of poor sleep quality among patients with established CHD in Kazakhstan who were assessed 6–24 months after their index coronary event, and (b) identify socio-demographic, behavioral, mental health, and clinical correlates of poor sleep quality, including potential sex-specific associations and interactions.

## 2. Materials and Methods

### 2.1. Study Design and Setting

This observational cross-sectional study was conducted from 1 October 2024 to 31 May 2025 in two geographical regions in Kazakhstan and two participating centers—the Research Institute of Cardiology and Internal Diseases in Almaty and the Medical Center of West Kazakhstan Medical University in Aktobe, both being major research centers in their regions with administrative capacity for participant recruitment. Participants were invited to attend a standardized study visit, including an interview and clinical examination, 6 to 24 months after the index coronary event.

### 2.2. Inclusion and Exclusion Criteria

Participants aged ≥ 18 with established coronary heart disease who had been hospitalized for an index event (STEMI, non-STEMI, or unstable angina) or had undergone an elective intervention procedure (elective percutaneous coronary intervention or elective coronary artery bypass grafting) within the 6–24-month period prior to recruitment were included in this study. Participants aged < 18, with severe physical disabilities, with a pre-existing clinical diagnosis of dementia or cognitive impairment, with other diagnoses, or with hospital admissions from outside the predefined period of 6–24 months were not eligible for participation in this study.

### 2.3. Study Population and Sample Size

The sample size was determined pragmatically from the recruitment capacity of the participating centers over the study period. Each center recruited 201 consecutively eligible patients, yielding a target of 402 participants.

### 2.4. Variables and Measures

The sleep quality levels were measured through the Pittsburgh Sleep Quality Index (PSQI), where a score > 5 was categorized as poor sleep quality and a score ≤ 5 was categorized as adequate sleep quality [42,43].

Sociodemographic variables included age (categorized as <60, ≥60), sex (categorized as males or females), ethnicity (categorized as Kazakhs/other Asians, European/Russians), marital status (categorized as widow/widower, never been married, divorced/separated, married), education (categorized as higher and primary education), living arrangement (categorized as alone or with somebody), social support availability (categorized as yes or no), living location (rural vs. urban/suburban), income level (categorized as low, middle, or high), index timing (acute or elective), attendance at a cardiac rehabilitation program (categorized as yes or no) and employment (during the past 6 months, categorized as retired or employed).

Obesity was categorized according to the NICE guideline through the calculation of body mass index (BMI categorized as obese ≥ 30 kg/m^2^, overweight 25 kg/m^2^ to 29.9 kg/m^2^, normal 18.5 kg/m^2^ to 24.9 kg/m^2^, underweight < 18.5 kg/m^2^) [44].

The PA levels were measured through the International Physical Activity Questionnaire Short Form (IPAQ-SF), with classification into low, moderate, or high levels based on MET scores, following the official scoring guidelines [45,46,47]. For our analysis, we categorized it as either Low PA or Moderate-to-Vigorous Physical Activity (MVPA).

Anxiety and depression were categorized with the Hospital Anxiety and Depression Scale (HADS), with cut-off scores ≤ 7 corresponding to “No depression or anxiety”, 8–10 to “Borderline abnormal”, and a score ≥ 11 defined as “Abnormal” [48,49]. “Borderline abnormal” and “Abnormal” values were merged in the logistic regression analysis.

The cognitive function was assessed through the Mini-MoCA tool [50]. The following categorization was implemented using the groups of “Normal cognitive skills” for scores of 26–30, “Mild cognitive impairment” for scores of 18–25, “Moderate cognitive impairment” for scores of 10–17, and “Severe cognitive impairment” for scores below 10. Cognitive impairment values were merged into one value of “Declined cognitive skills” in the logistic regression analysis.

Alcohol consumption was categorized as positive if the respondent answered “Yes” to questions regarding alcohol consumption, while “No” and “Past user” were categorized as negative results. Although a similar logic applied to smoking status, an additional objective clarification with a CO measurement device—Smokerlyzer was used [51]. A value > 6 changed the smoking status to positive regardless of the respondent’s answer.

Participants were classified as having type 2 diabetes if they had a documented diagnosis of type 2 diabetes or an HbA1c of 6.5% or higher, a 120 min oral glucose tolerance test of 11.1 mmol/L or higher, a random blood glucose of 11.1 mmol/L or higher, or a fasting blood glucose of 7.0 mmol/L or higher in the hospital discharge letter, or an affirmative self-reported diagnosis during the interview [52,53].

A status of arterial hypertension included a self-reported history of high blood pressure or an objective study measurement demonstrating an average resting systolic blood pressure (SBP) of 140 mmHg or higher or an average resting diastolic blood pressure (DBP) of 90 mmHg or higher, calculated from two consecutive measurements taken five minutes apart during the interview, or a documented historical measurement of SBP of 140 mmHg or higher or DBP of 90 mmHg or higher recorded in the discharge letter [54].

The first criterion for chronic kidney disease (CKD) was a documented medical history of the disease or an established estimated glomerular filtration rate corresponding to a clinical stage, as noted in the hospital discharge letter, while the second criterion was a self-reported diagnosis provided by the participant during the study interview.

Lastly, beta-blockers and diuretics, as the main medications for CHD that could alter sleep, were also assessed during the interview through participant self-report [55,56].

### 2.5. Statistical Analysis

All statistical analyses were conducted in R version 4.5.0. Categorical variables were expressed as frequencies and percentages. Between-group differences were evaluated using Pearson’s chi-squared test, and Fisher’s exact test was applied when more than 20% of cells had expected frequencies below five. Continuous variables were summarized as medians and interquartile ranges, while the group differences were assessed with the Wilcoxon rank sum test after a Shapiro–Wilk test check. To account for multiple testing across the descriptive comparisons, Benjamini–Hochberg false-discovery-rate (FDR)-adjusted *p*-values were additionally computed. Statistical significance was set at a two-sided α of 0.05.

Poor sleep quality, defined a priori as a PSQI score > 5, served as the binary outcome. Univariable (unadjusted) logistic regression was used to estimate the crude association of each socio-demographic, behavioral, psychological, and clinical variable with poor sleep. All candidate variables were then entered into a multivariable logistic regression model to obtain mutually adjusted odds ratios. To preserve statistical power and ensure adequate cell counts, the HADS “borderline abnormal” and “abnormal” categories were combined into a single positive (anxious/depressed) category, and all Mini-MoCA impairment levels were combined into a single “declined cognitive skills” category. Model performance was evaluated by multicollinearity (variance inflation factors, using the generalized ${VIF}^{\frac{1}{2\times Df}}$ for multi-level terms), discrimination (area under the receiver-operating-characteristic curve [AUC] with 95% CI), and calibration (Hosmer–Lemeshow goodness-of-fit test).

Post hoc exploratory subgroup analyses were undertaken by re-fitting the logistic models within strata defined by sex, physical activity level (low vs. moderate-to-vigorous), anxiety status, and age group (<60 vs. ≥60 years). Effect modification was then formally evaluated by introducing multiplicative interaction terms into the models. Given the number of subgroup comparisons, *p*-values from the stratified analyses were adjusted for multiple testing using the Benjamini–Hochberg FDR method, and subgroup findings were interpreted with corresponding caution. All odds ratios (OR) are reported with their corresponding 95% confidence intervals (CI).

## 3. Results

A total of 398 participants were included in the analysis, of whom 147 (36.9%) reported poor sleep quality, while the remaining 251 (63.1%) reported adequate sleep quality. The socio-demographic characteristics and the differences between groups are shown in Table 1. The poor-sleep group was more frequently observed among older patients, though this difference did not remain significant after FDR correction (*p* = 0.026, q = 0.110). A similar tendency was observed, with females reporting a higher prevalence of poor sleep quality (46.3% vs. 31.1%, *p* = 0.002, q = 0.022). Additionally, retirement status was strongly associated with poor sleep quality (78.9% vs. 62.5%, *p* < 0.001, q = 0.016). Marital status also differed significantly, with a higher prevalence of widows/widowers and divorced participants in the poor sleep group (*p* = 0.004, q = 0.028). The remaining socio-demographic characteristics, such as living location, income level, education level, ethnicity, and living status, showed no significant difference.

Behavioral risk factors and mental health characteristics are demonstrated in Table 2. Although cognitive skills did not show a significant difference, anxiety and depression were significantly more prevalent among those with poor sleep than among those with adequate sleep quality, with the exception that depression did not remain significant after FDR correction (*p* < 0.05, q = 0.137). Similarly, a low level of PA was more frequent in the poor sleep group than in the adequate sleep group; however, this difference was not significant after FDR correction (44.9% vs. 33.9%, *p* = 0.029, q = 0.110), while the remaining behavioral risk factors failed to show significant differences.

Regarding the non-communicable diseases (Table 3), none showed a significant difference according to sleep quality. Although no difference was observed for beta-blockers, diuretic use tended to be higher in participants with poor sleep (14.3% vs. 24.0%, *p* = 0.011); however, it did not retain significance after FDR correction (q = 0.060).

According to Table 4, the unadjusted logistic regression model analysis demonstrated that each five-year increase in age (OR 1.15, 95% CI: 1.02 to 1.29), being retired (OR 2.24, 95% CI: 1.41 to 3.63), the presence of anxiety (OR 2.12, 95% CI: 1.34 to 3.38), low PA (OR 1.59, 95% CI: 1.05 to 2.42), and diuretic usage (OR 1.94, 95% CI: 1.16 to 3.25) significantly increased the odds of poor sleep. Conversely, male sex (OR 0.52, 95% CI: 0.34 to 0.80) and being married (OR 0.46, 95% CI: 0.26 to 0.82) were protective factors.

In the adjusted model, only anxiety remained a statistically significant independent correlate of poor sleep quality (OR 1.88, 95% CI: 1.10 to 3.23). The remaining variables lost their associations. No multicollinearity was detected (all VIFs < 1.9, Figure S2). The model demonstrated adequate calibration (Hosmer–Lemeshow *p* = 0.083, Figure S3) and modest discrimination (AUC 0.691, 95% CI 0.638 to 0.744, Figure S4).

Further subgroup analysis by sex stratification showed that anxiety (OR 3.94, 95% CI: 1.66 to 10.0) and not having higher education (OR 2.58, 95% CI: 1.03 to 6.76) were strongly associated with poor sleep quality in females, but they were not significant in males. On the other hand, males who were retired (OR 2.27, 95% CI: 1.03 to 5.17) and had low PA (OR 2.31, 95% CI: 1.22 to 4.44) had higher odds of sleep quality, which was not observed in females (Table S2). Primary education was strongly associated with poor sleep quality in low PA participants in subgroup analysis by PA stratification (OR 2.77, 95% CI: 1.08 to 7.50), as well as anxiety in the MVPA group (OR 2.12, 95% CI: 1.03 to 4.39) (Table S3). Conversely, male sex was associated with lower odds (OR 0.36, 95% CI: 0.16 to 0.79) in the MVPA group. The subgroup analysis of anxiety status showed significance only for participants from Aktobe, with lower odds in the anxiety group (OR 0.20, 95% CI: 0.04 to 0.84) (Table S4). The subgroup analysis for participants aged <60 revealed that male sex was associated with lower odds (OR 0.22, 95% CI: 0.05 to 0.91), whereas alcohol consumption and diuretic usage were associated with higher odds (OR 4.31, 95% CI: 1.31 to 15.8 and OR 6.17, 95% CI: 1.19 to 34.9, respectively) (Table S5). Retirement status was associated with higher odds in the ≥60 years group (OR 2.64, 95% CI: 1.11, 6.92). No stratified association remained statistically significant after Benjamini–Hochberg or Bonferroni correction (all adjusted *p* > 0.05). Therefore, these subgroup findings are reported as exploratory.

The interaction analysis in Table S6 showed significant interactions, particularly between sex and anxiety, indicating that the association between anxiety and poor sleep was significantly weaker in men than in women (OR 0.29, 95% CI: 0.10 to 0.82).

## 4. Discussion

In this observational cross-sectional study, nearly one-third 36.9%) of subjects with established CHD presented with poor sleep quality, with anxiety being the single statistically significant independent factor associated with poor sleep quality. The results of the subgroup analysis demonstrated significant sex-based differences, with anxiety and lack of higher education as strong correlates in females, while retirement and low PA were strong correlates in males. Furthermore, the interaction analysis confirmed that the association between anxiety and poor sleep quality was significantly weaker in males compared to females.

The 36.9% prevalence of poor sleep quality observed in our cohort is broadly consistent with prior literature on patients with cardiovascular disease, although the range across studies is wide. A Chinese MINOCA study reported a 33.3% prevalence of poor sleep quality, very close to our estimate, and a 37.8% prevalence was reported among ambulatory cardiac patients in Ethiopia [33,57]. Larger Japanese and Italian cohorts, however, reported a 43–66% prevalence among hospitalized cardiovascular patients, and a systematic review of CHD patients found that nearly 70% of CAD populations may exceed the PSQI cut-off, with comorbid anxiety and depression conferring even worse sleep [32,34,58,59].

Importantly, while the timing was not significant in our assessment, it still might contribute to the prevalence we observed. Longitudinal studies show that sleep architecture and subjective sleep quality are most disturbed immediately after an acute coronary syndrome and improve substantially over the subsequent six months as patients transition out of the hospital environment [60]. However, in a 12-month longitudinal study of 180 ACS patients, approximately 50% continued to report sleep disturbance even one year after the event, indicating that a substantial subgroup did not fully recover spontaneously [61]. Likewise, a six-month follow-up study of 610 Chinese PCI patients found that sleep disturbance, anxiety, and depression frequently persisted alongside angina symptoms and were associated with adverse cardiovascular events [62].

The sex-specific patterns observed in our subgroup analysis also have precedent in international literature. Multiple studies and meta-analyses have shown that insomnia and poor sleep quality are more prevalent among women than men, and that the associations between sleep disturbance and psychological distress are often more pronounced in women [63,64,65,66,67]. Jono et al. specifically reported that the link between insomnia and depression was stronger among women than men in a large cardiovascular cohort [63], and a recent meta-analysis of CHD risk according to sleep duration found that the influence of sleep on cardiovascular risk differs significantly by sex, with shorter sleep being more harmful in women and longer sleep more harmful in men [68,69]. Hormonal differences, higher rates of mood disorders, greater pre-sleep worry, and gender-related psychosocial stressors have all been proposed to underlie women’s vulnerability to sleep disturbance [66,67]. The independent association of low PA with poor sleep in men is consistent with extensive evidence that regular physical activity improves PSQI scores, sleep latency, and sleep efficiency in older adults [70,71,72,73].

To our knowledge, this is the first multi-center cross-sectional study to examine sleep quality and its correlates among patients with established CHD in Kazakhstan and the wider Central Asian region, an under-represented setting for cardiovascular sleep research. We used internationally validated instruments—the PSQI for sleep quality [42,43], the Hospital Anxiety and Depression Scale (HADS) for anxiety and depression [49], and the IPAQ-SF for physical activity [46], which supports comparability with other international studies.

Several limitations should be acknowledged. First, the cross-sectional design precludes inferences about causality or temporal direction. Second, although the sleep quality assessment used a validated instrument—the PSQI —it was assessed only by self-report, which is a significant limitation. Hence, we did not perform polysomnography or actigraphy, and obstructive sleep apnea (OSA) was not formally screened using any validated instrument, which is recommended for future studies. Given the high prevalence and prognostic importance of OSA in CHD, unmeasured OSA may account for part of the poor sleep quality observed and confound its associations [74,75]. Incorporating OSA screening is a priority for future work in this population. The IPAQ-SF, although widely used and feasible, has only a modest correlation with accelerometer-derived measures and may misclassify activity levels [76,77]. Third, the rehabilitation was captured only as attendance (yes/no), so the number, intensity, or completion of sessions was not recorded, and dose–response effects of rehabilitation on sleep could not be examined. Fourth, our sample, recruited only from two major cities of Kazakhstan due to administrative and financial constraints, may not be fully representative of all CHD patients, and the proportion of female participants (36.7%)—although broadly consistent with the sex distribution of post-MI cohorts in the country—may have limited the power to detect smaller effects in female-only subgroups [7]. Finally, residual confounding cannot be excluded despite multivariable adjustment, and we did not assess all possible medication use (e.g., hypnotics or psychotropic drugs) that might independently affect sleep architecture in this analysis.

These findings have direct implications for secondary prevention in Kazakhstan and comparable Central Asian health systems. First, brief validated sleep and mood screening (e.g., PSQI and HADS) could be embedded into routine post-discharge and cardiac rehabilitation visits at low cost, allowing patients with poor sleep and anxiety to be identified and referred for cognitive-behavioral or pharmacological management. Second, the sex-specific pattern we observed prioritizes personal medicine—in women, screening and treatment pathways should prioritize anxiety and reach patients with lower educational attainment, who may face greater barriers to accessing care, whereas in men, promotion of physical activity and structured support around the transition to retirement may be more relevant. Third, because fewer than half of our patients had attended a rehabilitation program, strengthening referral to and capacity of cardiac rehabilitation, a natural setting for sleep and psychological screening, represents a concrete, actionable target for national secondary prevention policy.

## 5. Conclusions

In this multi-center cross-sectional study, more than one-third of patients with established coronary heart disease reported poor sleep quality 6–24 months after their index event. Anxiety emerged as the only independent associated factor in the adjusted analysis, and determinants of poor sleep may differ by sex, age, and physical activity status. These findings highlight poor sleep as a common and under-recognized problem in secondary prevention and suggest that routine screening for sleep disturbance and anxiety, together with sex, age, and physical activity-tailored interventions, may improve care for these populations. Prospective studies incorporating objective sleep measures are needed to confirm these associations and to evaluate whether targeting sleep and anxiety improves cardiovascular outcomes.

## Figures and Tables

**Table 1:** Socio-demographic characteristics of groups divided by the quality of sleep.

Variable	Adequate Sleep N = 251 ^1^	Poor Sleep N = 147 ^1^	Overall N = 398 ^1^	*p*-Value ^2^	q-Value ^3^
Age (years)	63 (58–70)	66 (60–72)	64 (58–71)	0.026	0.110
Age Group				0.050	0.137
<60 years	85 (33.9%)	36 (24.5%)	121 (30.4%)		
≥60 years	166 (66.1%)	111 (75.5%)	277 (69.6%)		
Sex				0.002	0.022
Female	78 (31.1%)	68 (46.3%)	146 (36.7%)		
Male	173 (68.9%)	79 (53.7%)	252 (63.3%)		
Living Location				0.633	0.777
Urban/suburban	229 (91.2%)	132 (89.8%)	361 (90.7%)		
Rural	22 (8.8%)	15 (10.2%)	37 (9.3%)		
Income Level				0.757	0.817
High	3 (1.2%)	2 (1.4%)	5 (1.3%)		
Low	10 (4.0%)	8 (5.4%)	18 (4.5%)		
Middle	238 (94.8%)	137 (93.2%)	375 (94.2%)		
Education Level				0.149	0.279
Higher education	93 (37.1%)	44 (29.9%)	137 (34.4%)		
Primary education	158 (62.9%)	103 (70.1%)	261 (65.6%)		
Ethnicity				0.628	0.777
Any other Asian background	184 (73.3%)	111 (75.5%)	295 (74.1%)		
White/Other	67 (26.7%)	36 (24.5%)	103 (25.9%)		
Working status				<0.001	0.016
Employed	94 (37.5%)	31 (21.1%)	125 (31.4%)		
Retired	157 (62.5%)	116 (78.9%)	273 (68.6%)		
Marital status				0.004	0.028
Widow/Widower	29 (11.6%)	30 (20.4%)	59 (14.8%)		
Divorced/Separated	14 (5.6%)	18 (12.2%)	32 (8.0%)		
Married	202 (80.5%)	97 (66.0%)	299 (75.1%)		
Never been married	6 (2.4%)	2 (1.4%)	8 (2.0%)		
Living status				0.224	0.356
Alone	17 (6.8%)	15 (10.2%)	32 (8.0%)		
With somebody	234 (93.2%)	132 (89.8%)	366 (92.0%)		
Social support	241 (96.0%)	140 (95.2%)	381 (95.7%)	0.711	0.817
Acute or elective				0.755	0.817
Acute	208 (82.9%)	120 (81.6%)	328 (82.4%)		
Elective	43 (17.1%)	27 (18.4%)	70 (17.6%)		
Months since the index event	9.0 (8.0–11.0)	9.0 (9.0–11.0)	9.0 (8.0–11.0)	0.118	0.279
Body mass index (kg/m^2^)	29.2 (26.2–31.8)	28.1 (25.8–31.6)	28.7 (26.0–31.7)	0.297	0.446

^1^ Mean (SD); n (%); ^2^ Wilcoxon rank sum test; Pearson’s Chi-squared test; Fisher’s exact test; ^3^ False discovery rate correction for multiple testing.

**Table 2:** Behavioral risk factors and mental health among sleep groups.

Variable	Adequate Sleep N = 251 ^1^	Poor Sleep N = 147 ^1^	Overall N = 398 ^1^	*p*-Value ^2^	q-Value ^3^
Attended a rehabilitation program	117 (46.6%)	67 (45.6%)	184 (46.2%)	0.842	0.846
Anxiety (HADS-A)				0.001	0.016
Abnormal	11 (4.4%)	19 (12.9%)	30 (7.5%)		
Borderline abnormal	38 (15.1%)	31 (21.1%)	69 (17.3%)		
Normal	202 (80.5%)	97 (66.0%)	299 (75.1%)		
Depression (HADS-D)				0.041	0.137
Abnormal	23 (9.2%)	9 (6.1%)	32 (8.0%)		
Borderline abnormal	29 (11.6%)	30 (20.4%)	59 (14.8%)		
Normal	199 (79.3%)	108 (73.5%)	307 (77.1%)		
Cognitive skills				0.384	0.519
Mild Cognitive Impairment (MCI)	171 (68.1%)	92 (63.0%)	263 (66.2%)		
Moderate Impairment	45 (17.9%)	36 (24.7%)	81 (20.4%)		
Normal	34 (13.5%)	17 (11.6%)	51 (12.8%)		
Severe Impairment	1 (0.4%)	1 (0.7%)	2 (0.5%)		
Alcohol consumption	63 (25.1%)	28 (19.0%)	91 (22.9%)	0.165	0.279
Smoking status				0.165	0.279
Non smoker	148 (59.0%)	97 (66.0%)	245 (61.6%)		
Smoker	103 (41.0%)	50 (34.0%)	153 (38.4%)		
PA level				0.029	0.110
MVPA	166 (66.1%)	81 (55.1%)	247 (62.1%)		
Low PA	85 (33.9%)	66 (44.9%)	151 (37.9%)		

^1^ n (%); ^2^ Pearson’s Chi-squared test; Fisher’s exact test; ^3^ False discovery rate correction for multiple testing.

**Table 3:** Non-communicable diseases among sleep groups.

Variable	Adequate Sleep N = 251 ^1^	Poor Sleep N = 147 ^1^	Overall N = 398 ^1^	*p*-Value ^2^	q-Value ^3^
Chronic kidney disease	58 (23.1%)	44 (29.9%)	102 (25.6%)	0.132	0.279
Diabetes type 2	102 (41.0%)	70 (48.6%)	172 (43.8%)	0.141	0.279
Arterial hypertension	225 (89.6%)	140 (95.2%)	365 (91.7%)	0.051	0.137
Overweight/Obese	206 (82.1%)	115 (78.2%)	321 (80.7%)	0.349	0.496
Beta-blocker use	190 (75.7%)	110 (74.8%)	300 (75.4%)	0.846	0.846
Diuretic use	36 (14.3%)	36 (24.5%)	72 (18.1%)	0.011	0.060

^1^ n (%); ^2^ Pearson’s Chi-squared test; Fisher’s exact test; ^3^ False discovery rate correction for multiple testing.

**Table 4:** Odds ratio of poor sleep based on respondents’ characteristics.

	Unadjusted OR				Adjusted OR		
Characteristic	N	OR	95% CI	*p*-Value	OR	95% CI	*p*-Value
**Age (per 5 years)**	398	1.15	1.02, 1.29	0.019	1.0	0.85, 1.17	0.948
**Sex**	398						
Female		—	—		—	—	
Male		0.52	0.34, 0.80	0.003	0.67	0.38, 1.18	0.164
**Living Location**	398						
Urban/suburban		—	—		—	—	
Rural		1.18	0.58, 2.34	0.634	0.91	0.40, 2.02	0.820
**Education Level**	398						
Higher education		—	—		—	—	
Primary education		1.38	0.89, 2.14	0.150	1.30	0.78, 2.19	0.317
**Working status**	398						
Employed		—	—		—	—	
Retired		2.24	1.41, 3.63	<0.001	1.75	0.94, 3.30	0.079
**Marital status**	398						
Widow/Widower		—	—		—	—	
Divorced/Separated		1.24	0.52, 2.98	0.622	1.61	0.61, 4.32	0.342
Married		0.46	0.26, 0.82	0.008	0.75	0.39, 1.46	0.395
Never been married		0.32	0.04, 1.53	0.186	0.38	0.05, 1.96	0.282
**Attended rehabilitation program**	398						
No		—	—		—	—	
Yes		0.96	0.64, 1.44	0.842	1.10	0.69, 1.75	0.701
**Ethnicity**	398						
Any other Asian background		—	—		—	—	
White/Other		0.89	0.55, 1.42	0.628	0.88	0.52, 1.46	0.617
**Anxiety (HADS-A)**	398						
Normal		—	—		—	—	
Anxious		2.12	1.34, 3.38	0.001	1.88	1.10, 3.23	0.021
**Depression (HADS-D)**	398						
Normal		—	—		—	—	
Depressed		1.38	0.85, 2.22	0.184	0.85	0.48, 1.49	0.576
**Cognitive Skills**	397						
Normal cognitive skills		—	—		—	—	
Declined cognitive skills		1.38	0.72, 2.75	0.344	1.20	0.58, 2.55	0.634
**Alcohol consumption**	398						
No		—	—		—	—	
Yes		0.70	0.42, 1.15	0.166	0.97	0.53, 1.74	0.911
**Smoking status**	398						
Non smoker		—	—		—	—	
Smoker		0.74	0.48, 1.13	0.165	1.09	0.63, 1.90	0.753
**PA level**	398						
MVPA		—	—		—	—	
Low PA		1.59	1.05, 2.42	0.029	1.43	0.90, 2.28	0.133
**Chronic kidney disease**	398						
No		—	—		—	—	
Yes		1.42	0.90, 2.25	0.133	1.16	0.66, 2.01	0.603
**Diabetes status**	393						
Normal		—	—		—	—	
Diabetes		1.36	0.90, 2.06	0.141	0.99	0.61, 1.60	0.974
**Arterial hypertension**	398						
No		—	—		—	—	
Yes		2.31	1.03, 5.90	0.056	1.91	0.79, 5.21	0.175
**Body mass index (per 1 kg/m^2^)**	398	1.00	0.96, 1.04	0.859	0.99	0.95, 1.04	0.741
**Beta-blocker use**	398						
No		—	—		—	—	
Yes		0.95	0.60, 1.54	0.846	0.83	0.48, 1.43	0.497
**Diuretic use**	398						
No		—	—		—	—	
Yes		1.94	1.16, 3.25	0.012	1.52	0.84, 2.75	0.167
**Months since the index event**	398	1.04	0.94, 1.14	0.463	1.05	0.93, 1.18	0.415
**Study center**	398						
Almaty		—	—		—	—	
Aktobe		1.08	0.72, 1.62	0.714	1.23	0.69, 2.21	0.489

Abbreviations: CI = Confidence Interval, OR = Odds Ratio.

## Data Availability

The data presented in this study are available on request from the corresponding author. The data are not publicly available due to privacy restrictions.

## Supplementary Materials

The following supporting information can be downloaded at: https://www.mdpi.com/article/10.3390/medsci14040439/s1, Table S1. Rationale and anticipated direction of association with poor sleep; Figure S1. Flowchart of participant inclusion in the analysis; Figure S2. Multicollinearity diagnostics for the multivariable logistic regression model; Figure S3. Calibration plot for the multivariable logistic regression model of poor sleep quality; Figure S4. Receiver-operating-characteristic (ROC) curve for the multivariable logistic regression model of poor sleep quality; Table S2: Odds ratio of poor sleep based on respondents characteristics stratified by sex; Table S3: Odds ratio of poor sleep based on respondents characteristics stratified by physical activity; Table S4: Odds ratio of poor sleep based on respondents characteristics stratified by anxiety status (HADS-A); Table S5: Odds ratio of poor sleep based on respondents characteristics stratified by age groups; Table S6: Odds ratio of poor sleep with variable interactions.

## Abbreviations

The following abbreviations are used in this manuscript:

ACS	Acute coronary syndrome
BMI	Body mass index
CABG	Coronary artery bypass grafting
CHD	Coronary heart disease
CI	Confidence interval
CKD	Chronic kidney disease
CO	Carbon monoxide
CVD	Cardiovascular disease
DBP	Diastolic blood pressure
HADS	Hospital Anxiety and Depression Scale
HADS-A	Hospital Anxiety and Depression Scale—Anxiety subscale
HADS-D	Hospital Anxiety and Depression Scale—Depression subscale
IPAQ-SF	International Physical Activity Questionnaire—Short Form
MET	Metabolic equivalent of task
MINOCA	Myocardial infarction with non-obstructive coronary arteries
Mini-MoCA	Mini Montreal Cognitive Assessment
MVPA	Moderate-to-vigorous physical activity
NCD	Non-communicable disease
NICE	National Institute for Health and Care Excellence
NSTEMI	Non-ST-elevation myocardial infarction
OR	Odds ratio
OSA	Obstructive sleep apnea
PA	Physical activity
PCI	Percutaneous coronary intervention
PSQI	Pittsburgh Sleep Quality Index
PTSD	Post-traumatic stress disorder
SBP	Systolic blood pressure
STEMI	ST-elevation myocardial infarction

## References

[1] Chong B., Jayabaskaran J., Jauhari S.M., Chan S.P., Goh R., Kueh M.T.W., Li H., Chin Y.H., Kong G., Anand V.V. (2025). Global Burden of Cardiovascular Diseases: Projections from 2025 to 2050. Eur. J. Prev. Cardiol..

[2] Di Cesare M., Perel P., Taylor S., Kabudula C., Bixby H., Gaziano T.A., McGhie D.V., Mwangi J., Pervan B., Narula J. (2024). The Heart of the World. Glob. Heart.

[3] Davletov D., Kulimbet M., Makhmutov A., Osser D., Pashimov M., Assembekov B., Davletov K. (2026). Burden of Ischemic Heart Disease in Central Asia from 1990 to 2021: A Systematic Analysis of the Global Burden of Disease Study 2021. Int. J. Environ. Res. Public Health.

[4] Stark B.A., DeCleene N.K., Desai E.C., Hsu J.M., Johnson C.O., Lara-Castor L., LeGrand K.E., Bhoomadevi A., Aalipour M.A., Aalruz H. (2025). Global, Regional, and National Burden of Cardiovascular Diseases and Risk Factors in 204 Countries and Territories, 1990-2023. JACC.

[5] Kassymkhan A., Ryskulova A.-G., Buribayeva Z., Nurmukhambetova B., Bizhanov K., Nabok D., Nassyrova N., Bapayeva M., Mirrakhimov E. (2026). Cardiovascular Disease Burden in Rural Central Asia: A Systematic Review of Epidemiological Trends and Mortality Patterns. Epidemiologia.

[6] Junusbekova G., Tundybayeva M., Akhtaeva N., Kosherbayeva L. (2023). Recent Trends in Cardiovascular Disease Mortality in Kazakhstan. Vasc. Health Risk Manag..

[7] Zhakhina G., Gaipov A., Salustri A., Gusmanov A., Sakko Y., Yerdessov S., Bekbossynova M., Abbay A., Sarria-Santamera A., Akbilgic O. (2023). Incidence, Mortality and Disability-Adjusted Life Years of Acute Myocardial Infarction in Kazakhstan: Data from Unified National Electronic Healthcare System 2014–2019. Front. Cardiovasc. Med..

[8] Laranjo L., Lanas F., Sun M.C., Chen D.A., Hynes L., Imran T.F., Kazi D.S., Kengne A.P., Komiyama M., Kuwabara M. (2024). World Heart Federation Roadmap for Secondary Prevention of Cardiovascular Disease: 2023 Update. Glob. Heart.

[9] Leon A.S., Franklin B.A., Costa F., Balady G.J., Berra K.A., Stewart K.J., Thompson P.D., Williams M.A., Lauer M.S. (2005). Cardiac Rehabilitation and Secondary Prevention of Coronary Heart Disease: An American Heart Association Scientific Statement from the Council on Clinical Cardiology (Subcommittee on Exercise, Cardiac Rehabilitation, and Prevention) and the Council on Nutrition, Physical Activity, and Metabolism (Subcommittee on Physical Activity), in Collaboration with the American Association of Cardiovascular and Pulmonary Rehabilitation. Circulation.

[10] De Bacquer D., Astin F., Kotseva K., Pogosova N., De Smedt D., De Backer G., Rydén L., Wood D., Jennings C., for the EUROASPIRE IV and V Surveys of the European Observational Research Programme of the European Society of Cardiology (2022). Poor Adherence to Lifestyle Recommendations in Patients with Coronary Heart Disease: Results from the EUROASPIRE Surveys. Eur. J. Prev. Cardiol..

[11] Kotseva K., De Bacquer D., Jennings C., Gyberg V., De Backer G., Rydénz L., Amouyel P., Bruthans J., Cifkova R., Deckers J.W. (2017). Time Trends in Lifestyle, Risk Factor Control, and Use of Evidence-Based Medications in Patients with Coronary Heart Disease in Europe: Results from 3 EUROASPIRE Surveys, 1999–2013. Glob. Heart.

[12] Kotseva K., Wood D., De Bacquer D., De Backer G., Rydén L., Jennings C., Gyberg V., Amouyel P., Bruthans J., Castro Conde A. (2016). EUROASPIRE IV: A European Society of Cardiology Survey on the Lifestyle, Risk Factor and Therapeutic Management of Coronary Patients from 24 European Countries. Eur. J. Prev. Cardiol..

[13] Kotseva K. (2017). on behalf of the EUROASPIRE Investigators. The EUROASPIRE Surveys: Lessons Learned in Cardiovascular Disease Prevention. Cardiovasc. Diagn. Ther..

[14] Lloyd-Jones D.M., Allen N.B., Anderson C.A.M., Black T., Brewer L.C., Foraker R.E., Grandner M.A., Lavretsky H., Perak A.M., Sharma G. (2022). Life’s Essential 8: Updating and Enhancing the American Heart Association’s Construct of Cardiovascular Health: A Presidential Advisory from the American Heart Association. Circulation.

[15] Kwok C.S., Kontopantelis E., Kuligowski G., Gray M., Muhyaldeen A., Gale C.P., Peat G.M., Cleator J., Chew-Graham C., Loke Y.K. (2018). Self-Reported Sleep Duration and Quality and Cardiovascular Disease and Mortality: A Dose-Response Meta-Analysis. J. Am. Heart Assoc..

[16] Huang Y.-M., Xia W., Ge Y.-J., Hou J.-H., Tan L., Xu W., Tan C.-C. (2022). Sleep Duration and Risk of Cardio-Cerebrovascular Disease: A Dose-Response Meta-Analysis of Cohort Studies Comprising 3.8 Million Participants. Front. Cardiovasc. Med..

[17] Wang S., Li Z., Wang X., Guo S., Sun Y., Li G., Zhao C., Yuan W., Li M., Li X. (2022). Associations between Sleep Duration and Cardiovascular Diseases: A Meta-Review and Meta-Analysis of Observational and Mendelian Randomization Studies. Front. Cardiovasc. Med..

[18] Cappuccio F.P., Cooper D., D’Elia L., Strazzullo P., Miller M.A. (2011). Sleep Duration Predicts Cardiovascular Outcomes: A Systematic Review and Meta-Analysis of Prospective Studies. Eur. Heart J..

[19] Clark A., Lange T., Hallqvist J., Jennum P., Rod N.H. (2014). Sleep Impairment and Prognosis of Acute Myocardial Infarction: A Prospective Cohort Study. Sleep.

[20] Condén E., Rosenblad A. (2016). Insomnia Predicts Long-Term All-Cause Mortality after Acute Myocardial Infarction: A Prospective Cohort Study. Int. J. Cardiol..

[21] Ali E., Shaikh A., Yasmin F., Sughra F., Sheikh A., Owais R., Raheel H., Virk H.U.H., Mustapha J.A. (2023). Incidence of Adverse Cardiovascular Events in Patients with Insomnia: A Systematic Review and Meta-Analysis of Real-World Data. PLoS ONE.

[22] Javaheri S., Redline S. (2017). Insomnia and Risk of Cardiovascular Disease. Chest.

[23] Frøjd L.A., Dammen T., Munkhaugen J., Weedon-Fekjær H., Nordhus I.H., Papageorgiou C., Sverre E. (2022). Insomnia as a Predictor of Recurrent Cardiovascular Events in Patients with Coronary Heart Disease. Sleep Adv..

[24] Sarode R., Nikam P.P. (2023). The Impact of Sleep Disorders on Cardiovascular Health: Mechanisms and Interventions. Cureus.

[25] Greenlund I.M., Carter J.R. (2022). Sympathetic Neural Responses to Sleep Disorders and Insufficiencies. Am. J. Physiol.-Heart Circ. Physiol..

[26] Kadoya M., Koyama H. (2019). Sleep, Autonomic Nervous Function and Atherosclerosis. Int. J. Mol. Sci..

[27] Fernandez-Mendoza J. (2025). Insomnia Phenotypes, Cardiovascular Risk and Their Link to Brain Health. Circ. Res..

[28] Peng A., Lin Z., Zhu C. (2022). Relationship of Psychiatric Disorders and Sleep Quality to Physical Symptoms in Coronary Artery Disease. J. Nerv. Ment. Dis..

[29] Howarth N.E., Miller M.A. (2024). Sleep, Sleep Disorders, and Mental Health: A Narrative Review. Heart Mind.

[30] Alvaro P.K., Roberts R.M., Harris J.K. (2013). A Systematic Review Assessing Bidirectionality between Sleep Disturbances, Anxiety, and Depression. Sleep.

[31] Chen F., Lin H., Zhang Y., Zhang Y., Chen L. (2024). The Mediating Role of Sleep Disturbance in the Relationship between Depression and Cardiovascular Disease. Front. Psychiatry.

[32] Matsuda R., Kohno T., Kohsaka S., Shiraishi Y., Katsumata Y., Hayashida K., Yuasa S., Takatsuki S., Fukuda K. (2021). Psychological Disturbances and Their Association with Sleep Disturbances in Patients Admitted for Cardiovascular Diseases. PLoS ONE.

[33] Getahun Y., Demissie W.R., Amare H. (2021). Sleep Quality among Cardiac Patients on Follow up at Jimma Medical Center, Southwestern Ethiopia. Sleep Sci..

[34] Matsuda R., Kohno T., Kohsaka S., Fukuoka R., Maekawa Y., Sano M., Takatsuki S., Fukuda K. (2017). The Prevalence of Poor Sleep Quality and Its Association with Depression and Anxiety Scores in Patients Admitted for Cardiovascular Disease: A Cross-Sectional Designed Study. Int. J. Cardiol..

[35] Cao X., Wu J., Gu Y., Liu X., Deng Y., Ma C. (2021). Post-Traumatic Stress Disorder and Risk Factors in Patients with Acute Myocardial Infarction After Emergency Percutaneous Coronary Intervention: A Longitudinal Study. Front. Psychol..

[36] Edmondson D., Richardson S., Falzon L., Davidson K.W., Mills M.A., Neria Y. (2012). Posttraumatic Stress Disorder Prevalence and Risk of Recurrence in Acute Coronary Syndrome Patients: A Meta-Analytic Review. PLoS ONE.

[37] Chong R.J., Hao Y., Tan E.W.Q., Mok G.J.L., Sia C.-H., Ho J.S.Y., Chan M.Y.Y., Ho A.F.W. (2025). Prevalence of Depression, Anxiety and Post-Traumatic Stress Disorder (PTSD) After Acute Myocardial Infarction: A Systematic Review and Meta-Analysis. J. Clin. Med..

[38] Zeng J., Qiu Y., Yang C., Fan X., Zhou X., Zhang C., Zhu S., Long Y., Hashimoto K., Chang L. (2025). Cardiovascular Diseases and Depression: A Meta-Analysis and Mendelian Randomization Analysis. Mol. Psychiatry.

[39] Lichtman J.H., Bigger J.T., Blumenthal J.A., Frasure-Smith N., Kaufmann P.G., Lespérance F., Mark D.B., Sheps D.S., Taylor C.B., Froelicher E.S. (2008). Depression and Coronary Heart Disease: Recommendations for Screening, Referral, and Treatment: A Science Advisory from the American Heart Association Prevention Committee of the Council on Cardiovascular Nursing, Council on Clinical Cardiology, Council on Epidemiology and Prevention, and Interdisciplinary Council on Quality of Care and Outcomes Research: Endorsed by the American Psychiatric Association. Circulation.

[40] Gan Y., Gong Y., Tong X., Sun H., Cong Y., Dong X., Wang Y., Xu X., Yin X., Deng J. (2014). Depression and the Risk of Coronary Heart Disease: A Meta-Analysis of Prospective Cohort Studies. BMC Psychiatry.

[41] Nicholson A., Kuper H., Hemingway H. (2006). Depression as an Aetiologic and Prognostic Factor in Coronary Heart Disease: A Meta-Analysis of 6362 Events among 146 538 Participants in 54 Observational Studies. Eur. Heart J..

[42] Buysse D.J., Reynolds C.F., Monk T.H., Berman S.R., Kupfer D.J. (1989). The Pittsburgh Sleep Quality Index: A New Instrument for Psychiatric Practice and Research. Psychiatry Res..

[43] Carpi M. (2025). The Pittsburgh Sleep Quality Index: A Brief Review. Occup. Med..

[44] National Institute for Health and Care Excellence (2026). Clinical Guidelines. Overweight and Obesity Management.

[45] Craig C.L., Marshall A.L., Sjöström M., Bauman A.E., Booth M.L., Ainsworth B.E., Pratt M., Ekelund U.L., Yngve A., Sallis J.F. (2003). International Physical Activity Questionnaire: 12-Country Reliability and Validity. Med. Sci. Sports Exerc..

[46] Booth M. (2000). Assessment of Physical Activity: An International Perspective. Res. Q. Exerc. Sport.

[47] Cheng H.L. (2016). A Simple, Easy-to-Use Spreadsheet for Automatic Scoring of the International Physical Activity Questionnaire (IPAQ) Short Form. https://www.researchgate.net/publication/310953872_A_simple_easy-to-use_spreadsheet_for_automatic_scoring_of_the_International_Physical_Activity_Questionnaire_IPAQ_Short_Form?channel=doi&linkId=583bbee208ae3a74b4a06f27&showFulltext=true.

[48] Montazeri A., Vahdaninia M., Ebrahimi M., Jarvandi S. (2003). The Hospital Anxiety and Depression Scale (HADS): Translation and Validation Study of the Iranian Version. Health Qual. Life Outcomes.

[49] Zigmond A.S., Snaith R.P. (1983). The Hospital Anxiety and Depression Scale. Acta Psychiatr. Scand..

[50] Granier K.L., Segal D.L. (2024). Convergent and Predictive Validity of the Mini MoCA and Considerations for Use among Older Adults. Psychiatry Res. Commun..

[51] Ramani V.K., Mhaske M., Naik R. (2023). Assessment of Carbon Monoxide in Exhaled Breath Using the Smokerlyzer Handheld Machine: A Cross-Sectional Study. Tob. Use Insights.

[52] Bajaj M., McCoy R.G., Balapattabi K., Bannuru R.R., Bellini N.J., Bennett A.K., Beverly E.A., Briggs Early K., ChallaSivaKanaka S., American Diabetes Association Professional Practice Committee for Diabetes (2026). 2. Diagnosis and Classification of Diabetes: Standards of Care in Diabetes—2026. Diabetes Care.

[53] Marx N., Federici M., Schütt K., Müller-Wieland D., Ajjan R.A., Antunes M.J., Christodorescu R.M., Crawford C., Di Angelantonio E., Eliasson B. (2023). 2023 ESC Guidelines for the Management of Cardiovascular Disease in Patients with Diabetes. Eur. Heart J..

[54] McEvoy J.W., McCarthy C.P., Bruno R.M., Brouwers S., Canavan M.D., Ceconi C., Christodorescu R.M., Daskalopoulou S.S., Ferro C.J., Gerdts E. (2024). 2024 ESC Guidelines for the Management of Elevated Blood Pressure and Hypertension. Eur. Heart J..

[55] Schweitzer P.K., Randazzo A.C. (2017). Drugs That Disturb Sleep and Wakefulness. Principles and Practice of Sleep Medicine.

[56] Ohishi M., Kubozono T., Higuchi K., Akasaki Y. (2021). Hypertension, Cardiovascular Disease, and Nocturia: A Systematic Review of the Pathophysiological Mechanisms. Hypertens. Res..

[57] Zhu C.-Y., Hu H.-L., Tang G.-M., Sun J.-C., Zheng H.-X., Zhai C.-L., He C.-J. (2022). Sleep Quality, Sleep Duration, and the Risk of Adverse Clinical Outcomes in Patients with Myocardial Infarction with Non-Obstructive Coronary Arteries. Front. Cardiovasc. Med..

[58] Lodi Rizzini F., Gómez-González A.M., Conejero-Cisneros R., Romero-Blanco M.J., Maldonado-Barrionuevo A., Salinas-Sánchez P., Jiménez-Navarro M. (2022). Effects of Cardiac Rehabilitation on Sleep Quality in Heart Disease Patients with and without Heart Failure. Int. J. Environ. Res. Public Health.

[59] Madsen M.T., Huang C., Zangger G., Zwisler A.D.O., Gögenur I. (2019). Sleep Disturbances in Patients with Coronary Heart Disease: A Systematic Review. J. Clin. Sleep Med..

[60] Schiza S.E., Simantirakis E., Bouloukaki I., Mermigkis C., Arfanakis D., Chrysostomakis S., Chlouverakis G., Kallergis E.M., Vardas P., Siafakas N.M. (2010). Sleep Patterns in Patients with Acute Coronary Syndromes. Sleep Med..

[61] von Känel R., Meister-Langraf R.E., Zuccarella-Hackl C., Schiebler S.L.F., Znoj H., Pazhenkottil A.P., Schmid J.-P., Barth J., Schnyder U., Princip M. (2022). Sleep Disturbance after Acute Coronary Syndrome: A Longitudinal Study over 12 Months. PLoS ONE.

[62] Liu X., Fowokan A., Grace S.L., Ding B., Meng S., Chen X., Xia Y., Zhang Y. (2021). Chinese Patients’ Clinical and Psychosocial Outcomes in the 6 Months Following Percutaneous Coronary Intervention. BMC Cardiovasc. Disord..

[63] Jono Y., Kohno T., Kohsaka S., Kitakata H., Shiraishi Y., Katsumata Y., Hayashida K., Yuasa S., Takatsuki S., Fukuda K. (2022). Sex Differences in Sleep and Psychological Disturbances among Patients Admitted for Cardiovascular Diseases. Sleep Breath..

[64] Bertisch S.M., Reid M., Lutsey P.L., Kaufman J.D., McClelland R., Patel S.R., Redline S. (2021). Gender Differences in the Association of Insomnia Symptoms and Coronary Artery Calcification in the Multi-Ethnic Study of Atherosclerosis. Sleep.

[65] Zeng L.-N., Zong Q.-Q., Yang Y., Zhang L., Xiang Y.-F., Ng C.H., Chen L.-G., Xiang Y.-T. (2020). Gender Difference in the Prevalence of Insomnia: A Meta-Analysis of Observational Studies. Front. Psychiatry.

[66] Boer J., Höhle N., Rosenblum L., Fietze I. (2023). Impact of Gender on Insomnia. Brain Sci..

[67] Benge E., Pavlova M., Javaheri S. (2024). Sleep Health Challenges among Women: Insomnia across the Lifespan. Front. Sleep.

[68] Li C., Luo S., Liang T., Song D., Fu J. (2025). Gender Correlation between Sleep Duration and Risk of Coronary Heart Disease: A Systematic Review and Meta-Analysis. Front. Cardiovasc. Med..

[69] Zhang B., Wang Y., Liu X., Zhai Z., Sun J., Yang J., Li Y., Wang C. (2021). The Association of Sleep Quality and Night Sleep Duration with Coronary Heart Disease in a Large-Scale Rural Population. Sleep Med..

[70] De Paz-Montón L.P., Carmona-Torres J.M., López-Fernández-Roldán Á., Molina-Madueño R.M., Navarrete-Tejero C., Laredo-Aguilera J.A. (2026). Physical Exercise Programmes to Improve Insomnia or Poor Sleep Quality in Non-Hospitalised Elderly People: A Systematic Review and Meta-Analysis. PeerJ.

[71] Xiong Z., Yuan Y., Qiu B., Yang Y., Bai Y., Wang J., Wang T., Liu H., ShangGuan Y., Jiang S. (2025). Optimal Exercise Type and Dose to Improve Sleep Quality in Older Adults: A Systematic Review and Network Meta-Analysis. BMC Geriatr..

[72] Vanderlinden J., Boen F., Van Uffelen J.G.Z. (2020). Effects of Physical Activity Programs on Sleep Outcomes in Older Adults: A Systematic Review. Int. J. Behav. Nutr. Phys. Act..

[73] Solis-Navarro L., Masot O., Torres-Castro R., Otto-Yáñez M., Fernández-Jané C., Solà-Madurell M., Coda A., Cyrus-Barker E., Sitjà-Rabert M., Pérez L.M. (2023). Effects on Sleep Quality of Physical Exercise Programs in Older Adults: A Systematic Review and Meta-Analysis. Clocks Sleep.

[74] Sanchez Sanchez C., Mora Robles J., Larrubia Valle J.I., Moncada Ventura A.M., Urbano Carrillo C.A. (2023). Sleep Quality and Sleep Disorders in Patients with Acute Coronary Syndrome. Eur. J. Prev. Cardiol..

[75] Maniaci A., Lavalle S., Parisi F.M., Barbanti M., Cocuzza S., Iannella G., Magliulo G., Pace A., Lentini M., Masiello E. (2024). Impact of Obstructive Sleep Apnea and Sympathetic Nervous System on Cardiac Health: A Comprehensive Review. J. Cardiovasc. Dev. Dis..

[76] Lee P.H., Macfarlane D.J., Lam T., Stewart S.M. (2011). Validity of the International Physical Activity Questionnaire Short Form (IPAQ-SF): A Systematic Review. Int. J. Behav. Nutr. Phys. Act..

[77] Balboa-Castillo T., Muñoz S., Serón P., Andrade-Mayorga O., Lavados-Romo P., Aguilar-Farias N. (2023). Validity and Reliability of the International Physical Activity Questionnaire Short Form in Chilean Adults. PLoS ONE.

