# Supplementary material for: Prevalence and Determinants of Poor Sleep Quality Among Patients with Established Coronary Heart Disease in Kazakhstan: A Multi-Center Cross-Sectional Study
Source: Med Sci (Basel). 2026 Jul 27;14(4):439. doi: 10.3390/medsci14040439 (PMC13515913; doi:10.3390/medsci14040439)
Supplement: Supplementary file 1 [file medsci-14-00439-s001.zip › medsci-4416026-supplementary.pdf]

Table S1. Rationale and anticipated direction of association with poor sleep.

Variable	Rationale for inclusion	Anticipated direction
Age (older)	Age-related sleep fragmentation, reduced slow-wave sleep	↑ odds
Female sex	Higher insomnia prevalence, stronger sleep–distress link	↑ odds
Lower education	Proxy for socioeconomic status, health literacy, stress exposure	↑ odds
Retirement	Loss of daily routine/structure, overlaps with age	↑ odds
Married	Social support, partnered routine	↓ odds
Income / location / ethnicity / living status / social support	Socioeconomic status and support gradients	Exploratory
Rehabilitation attendance	Lifestyle/psychological support	↓ odds
Anxiety (HADS-A)	Hyperarousal, pre-sleep worry. Bidirectional with sleep	↑ odds
Depression (HADS-D)	Disrupted sleep architecture. Bidirectional	↑ odds
Cognitive impairment	Neurodegenerative/circadian disruption	Exploratory
Low physical activity	PA improves sleep latency/efficiency	↑ odds
Smoking	Nicotine stimulant effect	↑ odds
Alcohol	Fragmented sleep architecture	↑ odds
Hypertension	Non-dipping BP, shared autonomic dysregulation	↑ odds
Diabetes	Nocturia, neuropathy, glucose dysregulation	↑ odds
Chronic kidney disease	Uremia, restless legs, metabolic disturbance	↑ odds
Higher BMI	OSA risk, metabolic/inflammatory pathways	↑ odds
Beta-blocker use	Melatonin suppression, insomnia, nightmares	↑ odds

Variable	Rationale for inclusion	Anticipated direction
Diuretic use	Nocturia-related awakenings	↑ odds
Time since index event	Sleep recovers over months post-event	↓ odds
Study center	Design covariate (two-site clustering)	No directional hypothesis

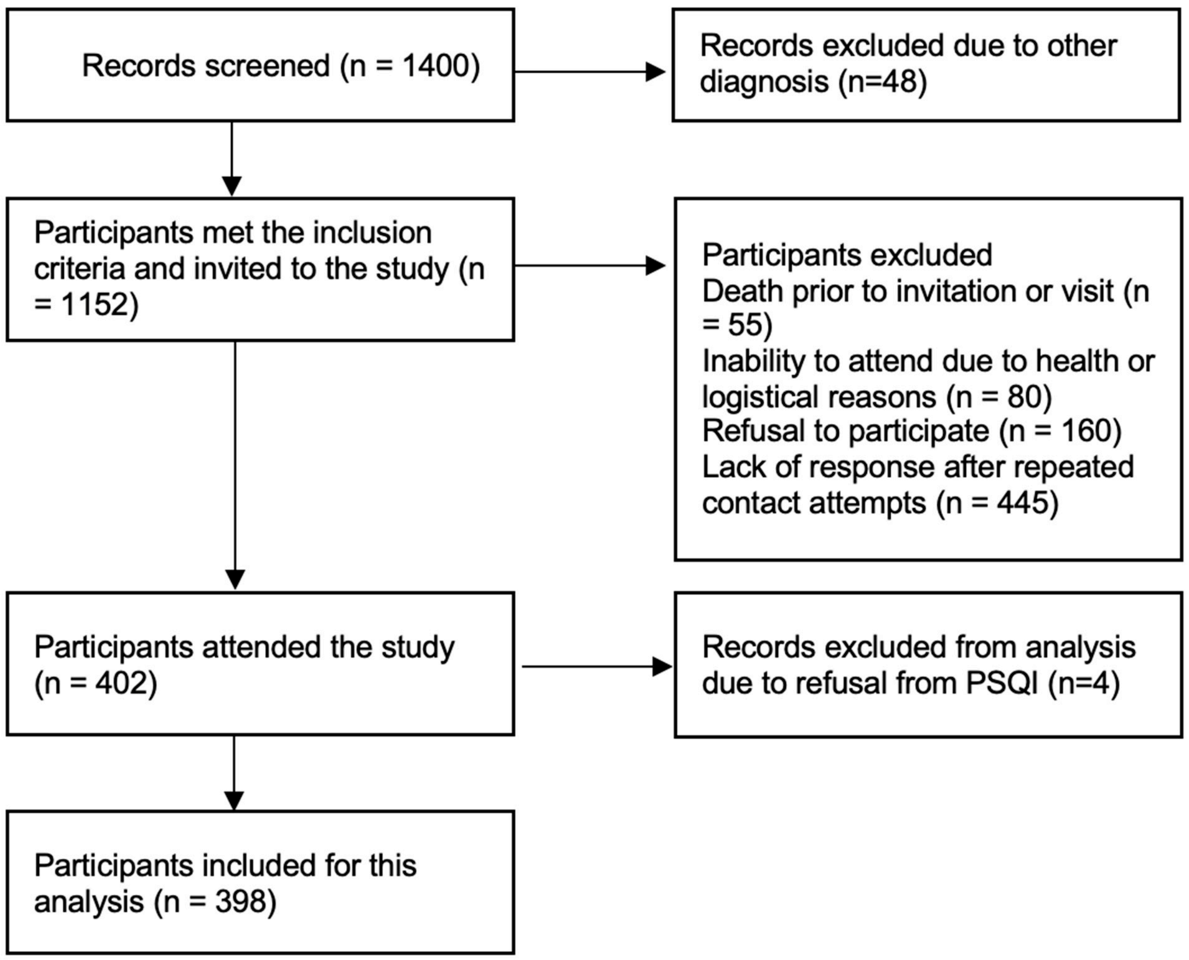

Figure S1. Flowchart of participant inclusion in the analysis.

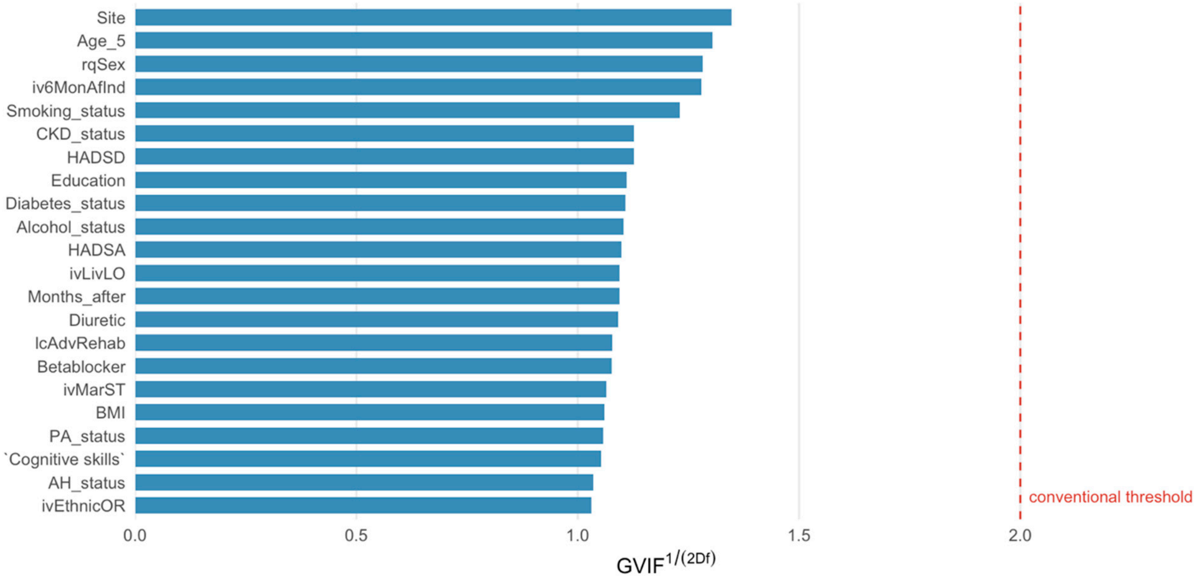

Figure S2. Multicollinearity diagnostics for the multivariable logistic regression model

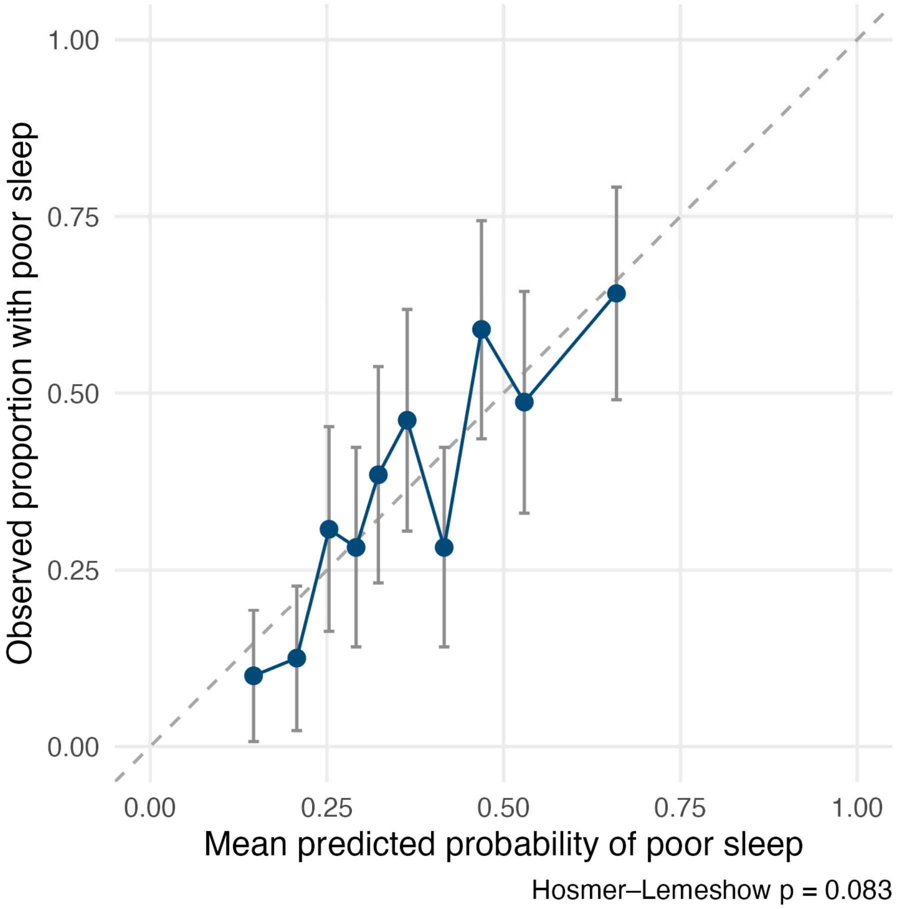

Figure S3. Calibration plot for the multivariable logistic regression model of poor sleep quality

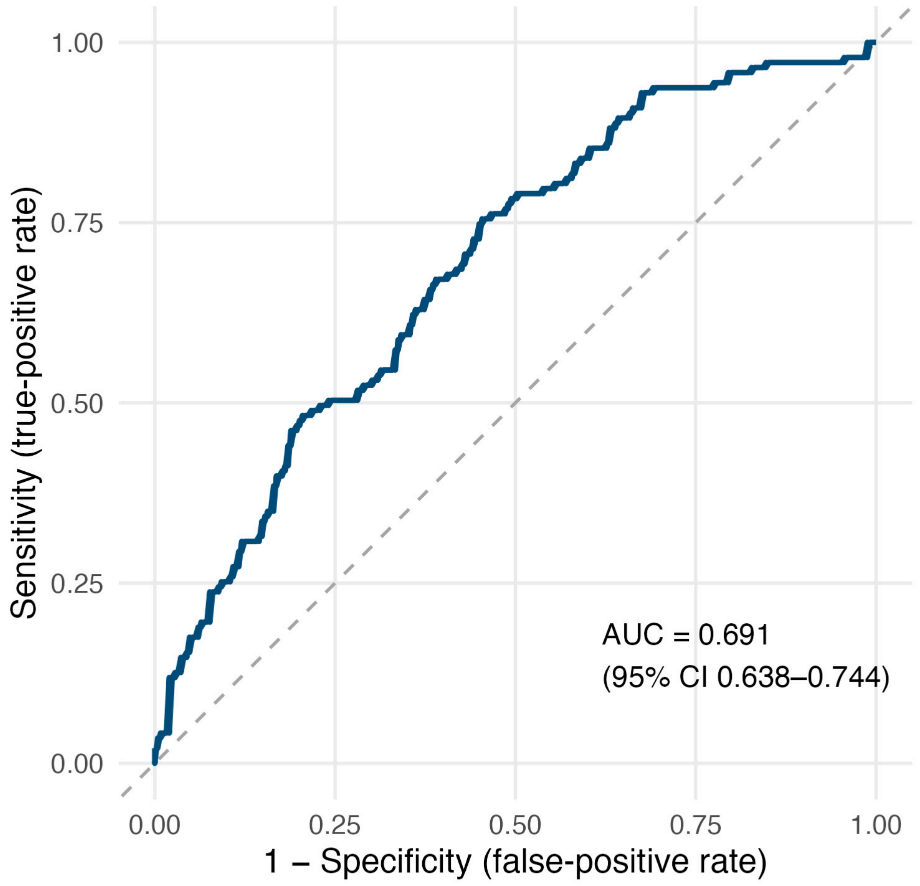

Figure S4. Receiver-operating-characteristic (ROC) curve for the multivariable logistic regression model of poor sleep quality

Table S2. Odds ratio of poor sleep based on respondents characteristics stratified by sex

Characteristic	Female			Male		
	OR	95% CI	p-value	OR	95% CI	p-value
**Age (per 5 years)**	0.94	0.69, 1.30	0.726	0.96	0.78, 1.19	0.713
**Living Location**						
Urban/ suburban	—	—		—	—	
Rural	0.76	0.21, 2.58	0.657	1.29	0.36, 4.25	0.684
**Education Level**						
Higher education	—	—		—	—	
Primary education	2.58	1.03, 6.76	0.047	1.06	0.53, 2.13	0.877
**Working status**						
Employed	—	—		—	—	
Retired	1.26	0.34, 4.77	0.727	2.27	1.03, 5.17	0.045
**Marital status**						
Widow/ Widower	—	—		—	—	
Divorced/ Separated	2.42	0.61, 10.6	0.222	0.83	0.14, 4.65	0.833
Married	1.31	0.49, 3.57	0.596	0.31	0.09, 1.00	0.053
Never been married	0.77	0.08, 6.35	0.809	0.00		0.985
**Attended rehabilitation program**						
No	—	—		—	—	
Yes	1.45	0.60, 3.56	0.411	0.96	0.51, 1.79	0.897
**Ethnicity**						
Any other Asian background	—	—		—	—	
White/Other	0.48	0.17, 1.28	0.150	1.21	0.61, 2.37	0.572
**Anxiety (HADS-A)**						
Normal	—	—		—	—	
Anxious	3.94	1.66, 10.0	0.003	1.06	0.46, 2.39	0.887

Characteristic	Female			Male		
	OR	95% CI	p-value	OR	95% CI	p-value
**Depression (HADS-D)**						
Normal	—	—		—	—	
Depressed	1.06	0.39, 2.79	0.913	0.69	0.30, 1.53	0.374
**Cognitive Skills**						
Normal cognitive skills	—	—		—	—	
Declined cognitive skills	0.59	0.17, 1.99	0.403	2.39	0.76, 9.65	0.170
**Alcohol consumption**						
No	—	—		—	—	
Yes	0.22	0.02, 1.59	0.174	1.19	0.61, 2.32	0.609
**Smoking status**						
Non smoker	—	—		—	—	
Smoker	0.24	0.04, 1.25	0.111	1.38	0.73, 2.67	0.324
**PA level**						
MVPA	—	—		—	—	
Low PA	0.85	0.37, 1.93	0.694	2.31	1.22, 4.44	0.011
**Chronic kidney disease**						
No	—	—		—	—	
Yes	1.42	0.56, 3.65	0.464	1.23	0.56, 2.68	0.597
**Diabetes status**						
Normal	—	—		—	—	
Diabetes	1.12	0.45, 2.76	0.811	1.02	0.53, 1.94	0.947
**Arterial hypertension**						
No	—	—		—	—	
Yes	2.72	0.31, 60.7	0.416	1.62	0.59, 5.06	0.373
**Body mass index (per 1 kg/m2)**	1.06	0.98, 1.16	0.161	0.98	0.92, 1.04	0.491
**Beta-blocker use**						
No	—	—		—	—	

Characteristic	Female OR	Female 95% CI	Female p-value	Male OR	Male 95% CI	Male p-value
Yes	0.82	0.29, 2.31	0.711	0.93	0.45, 1.93	0.840
**Diuretic use**						
No	—	—		—	—	
Yes	1.42	0.54, 3.82	0.476	2.01	0.85, 4.71	0.108
**Months since index event**	0.94	0.74, 1.16	0.602	1.15	0.99, 1.36	0.070
**Study center**						
Almaty	—	—		—	—	
Aktobe	0.90	0.29, 2.76	0.853	1.01	0.48, 2.16	0.973

Abbreviations: CI = Confidence Interval, OR = Odds Ratio

Table S3. Odds ratio of poor sleep based on respondents characteristics stratified by physical activity

Characteristic	MVPA			Low PA		
	OR	95% CI	p-value	OR	95% CI	p-value
**Age (per 5 years)**	1.11	0.90, 1.38	0.342	0.80	0.59, 1.07	0.132
**Sex**						
Female	—	—		—	—	
Male	0.36	0.16, 0.79	0.012	1.93	0.72, 5.42	0.198
**Living Location**						
Urban/ suburban	—	—		—	—	
Rural	1.07	0.33, 3.23	0.904	0.82	0.20, 3.40	0.788
**Education Level**						
Higher education	—	—		—	—	
Primary education	0.87	0.45, 1.71	0.689	2.77	1.08, 7.50	0.038
**Working status**						
Employed	—	—		—	—	
Retired	1.43	0.65, 3.20	0.380	2.69	0.85, 8.94	0.097
**Marital status**						
Widow/ Widower	—	—		—	—	
Divorced/ Separated	1.65	0.48, 5.76	0.425	2.22	0.32, 17.9	0.428
Married	0.86	0.35, 2.16	0.749	0.74	0.23, 2.42	0.618
Never been married	0.59	0.07, 3.65	0.582	0.00		0.984
**Attended rehabilitation program**						
No	—	—		—	—	
Yes	1.55	0.79, 3.07	0.203	0.79	0.35, 1.74	0.556
**Ethnicity**						
Any other Asian background	—	—		—	—	
White/Other	1.04	0.53, 2.02	0.904	0.66	0.25, 1.65	0.382

	MVPA			Low PA		
**Characteristic**	**OR**	**95% CI**	**p-value**	**OR**	**95% CI**	**p-value**
**Anxiety (HADS-A)**						
Normal	—	—		—	—	
Anxious	2.12	1.03, 4.39	0.042	2.15	0.82, 5.91	0.125
**Depression (HADS-D)**						
Normal	—	—		—	—	
Depressed	1.03	0.45, 2.29	0.940	0.59	0.23, 1.46	0.258
**Cognitive Skills**						
Normal cognitive skills	—	—		—	—	
Declined cognitive skills	0.95	0.39, 2.43	0.904	2.40	0.58, 11.8	0.246
**Alcohol consumption**						
No	—	—		—	—	
Yes	1.27	0.58, 2.75	0.544	0.65	0.22, 1.81	0.415
**Smoking status**						
Non smoker	—	—		—	—	
Smoker	1.49	0.69, 3.30	0.321	0.91	0.34, 2.44	0.846
**Chronic kidney disease**						
No	—	—		—	—	
Yes	1.12	0.53, 2.30	0.769	1.71	0.65, 4.66	0.283
**Diabetes status**						
Normal	—	—		—	—	
Diabetes	0.66	0.34, 1.26	0.215	1.56	0.64, 3.85	0.328
**Arterial hypertension**						
No	—	—		—	—	
Yes	1.53	0.56, 4.70	0.428	8.57	0.85, 247	0.121
**Body mass index (per 1 kg/m2)**	1.01	0.94, 1.07	0.857	0.97	0.89, 1.06	0.524
**Beta-blocker use**						
No	—	—		—	—	

	MVPA			Low PA		
**Characteristic**	**OR**	**95% CI**	**p-value**	**OR**	**95% CI**	**p-value**
Yes	0.87	0.43, 1.76	0.695	1.04	0.39, 2.83	0.936
**Diuretic use**						
No	—	—		—	—	
Yes	1.00	0.42, 2.33	0.995	2.48	0.94, 6.80	0.070
**Months since index event**	1.02	0.88, 1.17	0.833	1.21	0.96, 1.54	0.115
**Study center**						
Almaty	—	—		—	—	
Aktobe	1.57	0.72, 3.43	0.253	0.58	0.20, 1.66	0.315

Abbreviations: CI = Confidence Interval, OR = Odds Ratio

Table S4. Odds ratio of poor sleep based on respondents characteristics stratified by anxiety status (HADS-A)

Characteristic	Normal			Anxious		
	OR	95% CI	p-value	OR	95% CI	p-value
**Age (per 5 years)**	1.07	0.87, 1.31	0.533	0.83	0.58, 1.19	0.318
**Sex**						
Female	—	—		—	—	
Male	0.90	0.45, 1.84	0.781	0.31	0.08, 1.09	0.073
**Living Location**						
Urban/ suburban	—	—		—	—	
Rural	1.04	0.40, 2.58	0.938	1.52	0.17, 14.2	0.706
**Education Level**						
Higher education	—	—		—	—	
Primary education	1.54	0.84, 2.88	0.166	0.96	0.27, 3.34	0.942
**Working status**						
Employed	—	—		—	—	
Retired	2.07	0.97, 4.52	0.064	1.63	0.38, 7.33	0.513
**Marital status**						
Widow/ Widower	—	—		—	—	
Divorced/ Separated	2.61	0.71, 9.92	0.151	1.02	0.13, 8.77	0.983
Married	0.70	0.31, 1.61	0.398	0.79	0.16, 3.79	0.766
Never been married	0.52	0.06, 3.19	0.501	0.00		0.991
**Attended rehabilitation program**						
No	—	—		—	—	
Yes	1.02	0.57, 1.80	0.957	1.02	0.32, 3.23	0.967
**Ethnicity**						
Any other Asian background	—	—		—	—	
White/Other	1.10	0.58, 2.04	0.767	0.37	0.10, 1.31	0.129

Characteristic	Normal			Anxious		
	OR	95% CI	p-value	OR	95% CI	p-value
**Depression (HADS-D)**						
Normal	—	—		—	—	
Depressed	1.11	0.53, 2.28	0.785	1.01	0.31, 3.30	0.990
**Cognitive Skills**						
Normal cognitive skills	—	—		—	—	
Declined cognitive skills	1.33	0.55, 3.47	0.544	0.56	0.09, 3.25	0.515
**Alcohol consumption**						
No	—	—		—	—	
Yes	0.90	0.45, 1.78	0.773	1.45	0.25, 8.46	0.675
**Smoking status**						
Non smoker	—	—		—	—	
Smoker	1.40	0.74, 2.69	0.303	0.50	0.12, 2.04	0.334
**PA level**						
MVPA	—	—		—	—	
Low PA	1.45	0.82, 2.55	0.200	1.85	0.58, 6.30	0.308
**Chronic kidney disease**						
No	—	—		—	—	
Yes	1.41	0.70, 2.85	0.335	0.49	0.14, 1.58	0.240
**Diabetes status**						
Normal	—	—		—	—	
Diabetes	0.87	0.49, 1.54	0.637	2.79	0.79, 10.8	0.119
**Arterial hypertension**						
No	—	—		—	—	
Yes	1.37	0.51, 4.23	0.551	6.94	0.63, 195	0.160
**Body mass index (per 1 kg/m2)**	0.96	0.91, 1.02	0.217	1.07	0.96, 1.21	0.225
**Beta-blocker use**						
No	—	—		—	—	

Characteristic	Normal			Anxious		
	OR	95% CI	p-value	OR	95% CI	p-value
Yes	0.89	0.46, 1.73	0.722	0.61	0.16, 2.23	0.461
**Diuretic use**						
No	—	—		—	—	
Yes	1.46	0.72, 2.91	0.287	1.10	0.21, 5.77	0.909
**Months since index event**	1.04	0.91, 1.18	0.564	0.94	0.63, 1.41	0.773
**Study center**						
Almaty	—	—		—	—	
Aktobe	1.85	0.91, 3.82	0.091	0.20	0.04, 0.84	0.034

Abbreviations: CI = Confidence Interval, OR = Odds Ratio

Table S5. Odds ratio of poor sleep based on respondents characteristics stratified by age groups

Characteristic	<60 years			≥60 years		
	OR	95% CI	p-value	OR	95% CI	p-value
**Sex**						
Female	—	—		—	—	
Male	0.22	0.05, 0.91	0.041	0.89	0.45, 1.76	0.727
**Living Location**						
Urban/ suburban	—	—		—	—	
Rural	1.00	0.16, 5.18	0.997	0.86	0.30, 2.44	0.779
**Education Level**						
Higher education	—	—		—	—	
Primary education	1.03	0.31, 3.51	0.961	1.39	0.75, 2.62	0.297
**Working status**						
Employed	—	—		—	—	
Retired	1.51	0.45, 5.09	0.498	2.64	1.11, 6.92	0.036
**Marital status**						
Widow/ Widower	—	—		—	—	
Divorced/ Separated	0.00		0.990	3.32	1.00, 12.1	0.056
Married	0.00		0.990	0.78	0.38, 1.61	0.497
Never been married	0.00		0.987	0.82	0.10, 5.37	0.839
**Attended rehabilitation program**						
No	—	—		—	—	
Yes	0.58	0.20, 1.58	0.292	1.43	0.80, 2.60	0.232
**Ethnicity**						
Any other Asian background	—	—		—	—	
White/Other	0.88	0.26, 2.79	0.830	0.89	0.46, 1.68	0.718
**Anxiety (HADS-A)**						
Normal	—	—		—	—	

	<60 years			≥60 years		
**Characteristic**	**OR**	**95% CI**	**p-value**	**OR**	**95% CI**	**p-value**
Anxious	3.24	1.00, 10.9	0.052	1.95	0.99, 3.86	0.053
**Depression (HADS-D)**						
Normal	—	—		—	—	
Depressed	0.65	0.18, 2.23	0.508	0.86	0.42, 1.70	0.660
**Cognitive Skills**						
Normal cognitive skills	—	—		—	—	
Declined cognitive skills	4.55	0.94, 36.1	0.091	0.73	0.29, 1.84	0.500
**Alcohol consumption**						
No	—	—		—	—	
Yes	4.31	1.31, 15.8	0.020	0.53	0.24, 1.14	0.113
**Smoking status**						
Non smoker	—	—		—	—	
Smoker	1.54	0.45, 5.79	0.504	1.03	0.52, 2.05	0.925
**PA level**						
MVPA	—	—		—	—	
Low PA	1.71	0.61, 4.85	0.304	1.32	0.75, 2.35	0.336
**Chronic kidney disease**						
No	—	—		—	—	
Yes	0.93	0.22, 3.66	0.914	1.23	0.64, 2.38	0.531
**Diabetes status**						
Normal	—	—		—	—	
Diabetes	0.74	0.20, 2.55	0.637	1.07	0.60, 1.90	0.823
**Arterial hypertension**						
No	—	—		—	—	
Yes	0.60	0.13, 3.11	0.525	3.73	1.11, 17.3	0.053
**Body mass index (per 1 kg/m2)**	0.96	0.85, 1.07	0.446	1.01	0.95, 1.06	0.847

Characteristic	<60 years			≥60 years		
	OR	95% CI	p-value	OR	95% CI	p-value
**Beta-blocker use**						
No	—	—		—	—	
Yes	2.57	0.76, 9.82	0.143	0.56	0.28, 1.09	0.089
**Diuretic use**						
No	—	—		—	—	
Yes	6.17	1.19, 34.9	0.032	1.20	0.60, 2.36	0.608
**Months since index event**	1.08	0.84, 1.38	0.510	1.09	0.94, 1.26	0.261
**Study center**						
Almaty	—	—		—	—	
Aktobe	0.82	0.22, 3.00	0.765	1.67	0.81, 3.51	0.170

Abbreviations: CI = Confidence Interval, OR = Odds Ratio

Table S6. Odds ratio of poor sleep with variable interactions

	Sex * Anxiety			Sex * Physical Activity			Sex * Working Status			PA * Education		
**Characteristic**	**OR**	**95% CI**	**p-value**	**OR**	**95% CI**	**p-value**	**OR**	**95% CI**	**p-value**	**OR**	**95% CI**	**p-value**
**Age (per 5 years)**	0.98	0.84, 1.15	0.835	0.98	0.84, 1.16	0.853	0.99	0.85, 1.17	0.944	0.99	0.85, 1.16	0.926
**Sex**												
Female	—	—		—	—		—	—		—	—	
Male	0.95	0.50, 1.81	0.867	0.48	0.24, 0.96	0.039	0.42	0.15, 1.21	0.103	0.66	0.37, 1.16	0.149
**Living Location**												
Urban/ suburban	—	—		—	—		—	—		—	—	
Rural	0.93	0.40, 2.08	0.856	0.94	0.41, 2.09	0.877	0.88	0.39, 1.97	0.762	0.90	0.39, 2.01	0.803
**Education Level**												
Higher education	—	—		—	—		—	—		—	—	
Primary education	1.33	0.79, 2.25	0.284	1.33	0.80, 2.25	0.277	1.35	0.80, 2.28	0.263	1.02	0.54, 1.92	0.959
**Working status**												
Employed	—	—		—	—		—	—		—	—	
Retired	1.89	1.01, 3.58	0.049	1.78	0.95, 3.36	0.073	1.13	0.41, 3.21	0.809	1.77	0.95, 3.33	0.075
**Marital status**												
Widow/ Widower	—	—		—	—		—	—		—	—	
Divorced/ Separated	1.73	0.64, 4.75	0.284	1.51	0.57, 4.07	0.408	1.54	0.58, 4.15	0.384	1.62	0.61, 4.37	0.332
Married	0.75	0.38, 1.47	0.392	0.73	0.38, 1.42	0.349	0.72	0.37, 1.40	0.324	0.77	0.40, 1.50	0.439
Never been married	0.39	0.05, 2.02	0.292	0.31	0.04, 1.64	0.199	0.38	0.05, 1.97	0.283	0.38	0.05, 1.96	0.281
**Attended rehabilitation program**												
No	—	—		—	—		—	—		—	—	
Yes	1.07	0.67, 1.72	0.784	1.10	0.69, 1.76	0.699	1.08	0.68, 1.74	0.740	1.11	0.69, 1.77	0.677

	Sex * Anxiety			Sex * Physical Activity			Sex * Working Status			PA * Education		
**Characteristic**	**OR**	**95% CI**	**p-value**	**OR**	**95% CI**	**p-value**	**OR**	**95% CI**	**p-value**	**OR**	**95% CI**	**p-value**
**Ethnicity**												
Any other Asian background	—	—		—	—		—	—		—	—	
White/Other	0.87	0.51, 1.45	0.595	0.86	0.51, 1.44	0.578	0.87	0.52, 1.45	0.600	0.86	0.51, 1.44	0.568
**Anxiety (HADS-A)**												
Normal	—	—		—	—		—	—		—	—	
Anxious	3.65	1.67, 8.23	0.001	1.87	1.09, 3.21	0.023	1.84	1.07, 3.16	0.027	1.85	1.08, 3.17	0.026
**Depression (HADS-D)**												
Normal	—	—		—	—		—	—		—	—	
Depressed	0.89	0.49, 1.57	0.682	0.84	0.47, 1.47	0.546	0.85	0.48, 1.50	0.587	0.84	0.47, 1.48	0.544
**Cognitive Skills**												
Normal cognitive skills	—	—		—	—		—	—		—	—	
Declined cognitive skills	1.16	0.56, 2.51	0.692	1.15	0.55, 2.46	0.716	1.28	0.61, 2.76	0.523	1.21	0.59, 2.58	0.618
**Alcohol consumption**												
No	—	—		—	—		—	—		—	—	
Yes	0.96	0.53, 1.73	0.898	0.98	0.54, 1.77	0.946	0.98	0.54, 1.76	0.942	0.95	0.52, 1.72	0.879
**Smoking status**												
Non smoker	—	—		—	—		—	—		—	—	
Smoker	1.11	0.64, 1.93	0.716	1.10	0.63, 1.92	0.740	1.10	0.64, 1.92	0.728	1.12	0.64, 1.95	0.696
**PA level**												
MVPA	—	—		—	—		—	—		—	—	
Low PA	1.49	0.93, 2.39	0.101	0.90	0.44, 1.86	0.781	1.42	0.89, 2.27	0.141	0.91	0.39, 2.07	0.823

Characteristic	Sex * Anxiety			Sex * Physical Activity			Sex * Working Status			PA * Education		
	OR	95% CI	p-value	OR	95% CI	p-value	OR	95% CI	p-value	OR	95% CI	p-value
**Chronic kidney disease**												
No	—	—		—	—		—	—		—	—	
Yes	1.19	0.68, 2.08	0.542	1.17	0.67, 2.04	0.570	1.16	0.67, 2.02	0.592	1.16	0.66, 2.01	0.602
**Diabetes status**												
Normal	—	—		—	—		—	—		—	—	
Diabetes	0.99	0.61, 1.60	0.962	1.01	0.62, 1.63	0.980	1.00	0.62, 1.62	0.992	0.98	0.60, 1.58	0.930
**Arterial hypertension**												
No	—	—		—	—		—	—		—	—	
Yes	1.83	0.76, 4.98	0.205	1.90	0.78, 5.22	0.180	1.96	0.81, 5.40	0.159	1.90	0.79, 5.18	0.177
**Body mass index (per 1 kg/m2)**	0.99	0.94, 1.04	0.714	1.00	0.95, 1.04	0.836	0.99	0.95, 1.04	0.718	0.99	0.95, 1.04	0.785
**Beta-blocker use**												
No	—	—		—	—		—	—		—	—	
Yes	0.84	0.49, 1.46	0.534	0.85	0.49, 1.46	0.549	0.83	0.48, 1.43	0.499	0.84	0.49, 1.45	0.529
**Diuretic use**												
No	—	—		—	—		—	—		—	—	
Yes	1.48	0.81, 2.69	0.203	1.51	0.83, 2.74	0.174	1.53	0.84, 2.77	0.163	1.44	0.79, 2.62	0.235
**Months since index event**	1.06	0.94, 1.19	0.345	1.06	0.94, 1.19	0.335	1.05	0.94, 1.18	0.394	1.05	0.94, 1.18	0.401
**Study center**												
Almaty	—	—		—	—		—	—		—	—	
Aktobe	1.16	0.64, 2.10	0.617	1.18	0.65, 2.13	0.587	1.24	0.69, 2.22	0.479	1.22	0.68, 2.21	0.498
**Sex * Anxiety (HADS-A)**												
Male * Anxious	0.29	0.10, 0.82	0.021									

	**Sex * Anxiety**			**Sex * Physical Activity**			**Sex * Working Status**			**PA * Education**		
**Characteristic**	**OR**	**95% CI**	**p-value**	**OR**	**95% CI**	**p-value**	**OR**	**95% CI**	**p-value**	**OR**	**95% CI**	**p-value**
**Sex * PA level**												
Male * Low PA				2.17	0.86, 5.52	0.101						
**Sex * Working status**												
Male * Retired							1.83	0.58, 5.69	0.298			
**Education Level * PA level**												
Primary education * Low PA										1.94	0.72, 5.31	0.191

Abbreviations: CI = Confidence Interval, OR = Odds Ratio
